# How Does the Design of Consultation Pages Affect Patients’ Perception of Physician Authority and Willingness to Seek Offline Treatment: A Randomized Controlled Trial

**DOI:** 10.3390/bs13070584

**Published:** 2023-07-13

**Authors:** Qi Wang, Hao Wang, Si Wang, Wen Zhang

**Affiliations:** 1 School of Industrial Design, Hubei University of Technology, Wuhan 430068, China; wq20201103@hbut.edu.cn (Q.W.);; 2 School of Humanities, Jianghan University, Wuhan 430056, China; 3 School of Journalism and Culture Communication, Zhongnan University of Economics and Law, Wuhan 430073, China; z0004810@zuel.edu.cn

**Keywords:** online clinic, offline visits, dialogue box, consultation page design, physician authority, embodied cognition

## Abstract

This study aimed to assess the impact of the color and font size of a dialogue box on an online physician–patient interaction page on patients’ perceptions of the physician’s authority and their willingness to schedule an offline appointment. A 2 × 2 between-group experiment was conducted to compare the effects of two dialogue box colors (gold vs. grey) and two font sizes (large vs. regular) on patients’ perceptions. The results showed that a larger font size had a significant positive impact on patients’ perceptions of the physician’s authority, and the use of a gold-colored dialogue box also had a significant positive effect. A significant interaction was found between the dialogue box color and font size and patients’ perceptions of the physician’s authority. In addition, it was found that positive perceptions of the physician’s authority significantly affected patients’ willingness to schedule offline appointments and played a fully mediating role in the path of page design affecting offline appointment intentions. This study provides evidence that the design elements of a dialogue box—particularly, its color and font size—can influence patients’ perceptions of a physician’s authority and their willingness to schedule an offline appointment. These findings suggest that modifying the page design could improve the effectiveness of physician–patient communication.

## 1. Introduction

In the rapidly evolving digital age, the way we access and utilize healthcare services has undergone a significant transformation [1,2]. With the influx of innovative technologies, the internet has emerged as a powerful conduit for the exchange of medical information between physicians and patients [3]. Today, an increasing number of people are readily embracing online sources to acquire medical advice, fundamentally redefining the traditional healthcare delivery model [4,5].

Online healthcare platforms, or online clinics, are at the forefront of this transformative movement. Serving as the digital equivalent of a physical clinic, they have become an invaluable nexus for patients seeking medical services and health information [6,7]. By leveraging the power of digital connectivity, these platforms provide a comprehensive and immersive healthcare experience, going beyond the scope of traditional clinical interactions [8,9].

Online clinics significantly enhance the accessibility and convenience of medical consultations, extending the reach of healthcare services beyond geographical and temporal constraints. Patient-oriented design is especially important in this fast-paced society, addressing the most common issues of patient wait times, physician availability/overtime, and patient congestion [10]. They present a cost-effective alternative to traditional healthcare, eliminating the need for physical transportation and other out-of-home expenses [9,11]. Moreover, they offer a suite of digital services, including but not limited to graphical consultations, digital appointments, and instant access to test results and reports [12].

In the context of healthcare incorporating the Internet of Things, patient-oriented design will address the limited time, attention, and accuracy of people and provide real-time convenience for patients in collecting data about things in the real world [13]. These platforms serve as an interactive digital interface, enabling patients to remotely interact with their physicians. They offer highly specialized and personalized medical services and health information, allowing patients to receive customized treatment plans based on their unique medical history and test results [14,15]. As an indicator of their rising popularity, as of December 2022, the number of individuals using online healthcare services in China reached a staggering 363 million, marking an increase of 64.66 million or 34.0% of the total Internet population compared to December 2021 [16].

With their ever-growing prevalence, online clinics have evolved to serve a dual role. On one hand, they assist patients in identifying appropriate medical professionals based on their unique healthcare needs. On the other hand, they provide a platform for medical practitioners to convert virtual interactions into physical appointments, bridging the gap between digital and physical healthcare [17].

A patient’s willingness to transition from an online to an offline appointment is subject to a range of factors. These include the quality and depth of information available about the physician on the consultation platform, the level of trust established through online physician–patient communication, and the evaluations and assessments provided by previous patients [18,19].

While previous research has delved into the impact of textual information on patients’ decision-making processes during online consultations [20], there exists a knowledge gap. Limited studies have examined the relationship between the design elements of the online consultation platform, such as the page design, text size, and color, and patients’ inclination to schedule an offline appointment. This research endeavor aims to fill this gap, exploring the potential impact of such design features on patient perception and behavior.

Recognizing the design of the consultation page as a critical tool for creating a sense of ritual is vital [21]. As the direct interface for patients, the design can significantly influence their perceptions, emotions, and actions [22]. A well-structured and aesthetically pleasing design, featuring high saturation and warm-toned images, a consistent font and font size, and bright colors, can have a calming effect on the viewer, eliciting a positive response [23,24,25,26,27].

The authority of a physician, as perceived by a patient, is a pivotal factor influencing patient behaviors [28]. This perception is constructed by a combination of factors, including the physician’s professional and academic credentials, service attitude, number of services provided, and patient comments [29]. In this regard, the research also intends to investigate how the consultation page design impacts the patient’s perception of the physician’s authority and, subsequently, their intent to seek medical advice offline.

Consequently, this research undertook the task of constructing a structural equation model, employing embodied cognition as the theoretical pillar, within the scope of online medical care. The study subsequently scrutinized the impact of the consultation page’s design on patients’ perception of a physician’s authority and their inclination towards seeking offline treatment through the implementation of a randomized controlled trial. Through this comprehensive exploration, the research seeks to offer actionable insights for designing effective online consultation platforms, ultimately fostering the wider acceptance and utilization of telemedicine services.

## 2. Theoretical Foundation

### 2.1. Embodied Cognition

Embodied cognition theory posits that our cognitive processes, including perception, understanding, and decision making, are profoundly influenced by bodily sensations and experiences [30,31]. This theory fundamentally challenges the traditional cognitive science view that cognition is an abstract mental process isolated from physical and sensory experiences. Instead, it emphasizes a holistic view, proposing a tight interconnection between the mind and the body [32].

One of the critical perspectives of embodied cognition theory is that our understanding of abstract concepts is derived from, and thus metaphorically linked to, our physical and sensory experiences [33]. Lakoff and Johnson argued that these metaphorical associations play an essential role in our ability to comprehend abstract ideas and concepts, grounding them in our tangible experiences and interactions [34].

### 2.2. Embodied Cognition and Perception of Authority

The perception of authority is a complex cognitive process that is influenced by several factors, including professional credentials, evaluations by others, and professional experience [35]. According to embodied cognition theory, this perception is not just a mental construct but could be influenced by sensory experiences. In the context of online consultations, the physical properties of the digital interface, such as the font size and dialogue box color, could potentially shape the perceived authority of a physician.

Font size has been identified as a critical element in shaping perceptions of importance and credibility. Research by Meier et al. [36] demonstrated that the physical size of words can affect their evaluation. Similarly, Sorokowski et al. [37] found that individuals attribute a higher status and greater intelligence to authors of texts written in larger fonts. Extrapolating from these findings, it is plausible that a larger font size used in a dialogue box could enhance the perception of a physician’s authority.

The influence of color on cognition and decision making is also widely documented [38,39]. In the context of digital interfaces, color can significantly impact user experience, influencing perceptions and emotions [40,41]. For example, in the context of traditional Chinese culture, different colors represent different ranks and meanings. Gold was the imperial color, and royal buildings used gold and yellow tones, while commoner buildings could only use grey and white tones. Making gold rather than grey tends to make people perceive authority. In addition, the media often employ bright and shiny colors to evoke the image of a leader and to highlight their authority in people’s minds [42]. Evidence has shown that utilizing high saturation and warm-toned images can have a calming effect on the viewer [22], while incorporating brighter colors in the design elements can elicit more favorable responses compared to darker hues [23]. Song et al.’s [43] research found a metaphorical association between color and emotion, showing that warm colors (e.g., red and yellow) were associated with positive emotions. Similarly, Sherman’s study [44] suggested that color associations could influence moral judgments. The warm color (gold) and the cool color (grey) are more suitable for the application and research background of the embodied theory of physician–patient interaction interfaces; therefore, we choose “gold and grey” as the background color of the dialogue box. In an online consultation scenario, we try to demonstrate that the color of the dialogue box plays a key role in shaping the patient’s perception of the physician’s authority.

### 2.3. Physician Authority: A Mediating Factor

The influence of the dialogue box design on the patient’s decision to schedule an appointment can be mediated by the patients’ perceptions of the physician’s authority. In an online consultation scenario, the perceived authority is constructed through various factors, including the physician’s credentials, service attitude, and patient feedback [29]. In the area of healthcare, physician authority refers to the combined power and ability of a physician’s medical consultations, and it is the influence of recognition, respect, and trust generated by society and individuals [45]. The therapeutic definition of the professional role encourages the acceptance of this authority [46]. A high perception of physician authority leads to a higher level of patient compliance in physician consultations, positive trust in the physician, and positive psychological comfort for the patient himself, thus enhancing treatment outcomes [47].

These factors, when presented effectively using the right design elements (like font size and color), may enhance the patient’s perception of authority, thereby influencing the patient’s decision to schedule an appointment. Therefore, we hypothesize that the patient’s perception of a physician’s authority plays a mediating role, bridging the gap between the sensory experiences invoked by the dialogue box design and the patient’s decision-making process.

### 2.4. Building a Structural Equation Model

With this theoretical background, we propose a structural equation model for investigating the potential impact of these concrete sensory experiences on abstract patient perceptions and decision-making processes. In our model, the text size in the dialogue box is considered as an independent variable, and the color of the dialogue box is considered as a moderating variable. The physician’s authority felt by patients is posited to play a mediating role in the relationship between these sensory experiences and the patient’s willingness to seek offline medical treatment, which is the dependent variable.

The model thus provides a theoretical framework for understanding how the design of online consultation platforms might influence patient behavior. Through the lens of embodied cognition, it suggests that adjusting the text size and dialogue box color could be effective ways to influence patient perceptions of physician authority and their willingness to schedule offline appointments.

Future research can validate and refine this model, contributing to the development of more effective and user-friendly online healthcare platforms. This approach not only bridges the gap between theory and practice but also provides a robust scientific foundation for future design and technological enhancements in the realm of telemedicine.

## 3. Methodology

### 3.1. Research Participants

The recruitment of participants was carried out through a public university in central China via the use of a WeChat corporate platform. Invitations to participate in the study were disseminated through this platform. To ensure the suitability of the participants, a two-minute online survey was conducted to assess eligibility, based on the following criteria: (1) regular smartphone usage, (2) absence of any significant medical conditions, (3) major in a non-medical field unrelated to medicine, and (4) absence of immediate relatives in the medical profession. A total of 240 eligible participants were recruited, and each participant was compensated financially for their participation and provided informed consent prior to participation in the study.

### 3.2. Study Design

The experiment was conducted in a multimedia classroom, with participants seated at a suitable distance apart to minimize potential interference and facilitate individual focus. The participants were informed of the purpose of the experiment, which was to assess the functionality of an online medical platform. Access to the experiment was facilitated via smartphone QR code scanning, and the participants were first presented with a guideline page, in which they were instructed to imagine a scenario where they sought medical consultation for a skin issue. Subsequently, they were directed to the link page for the formal experiment, where they were randomly assigned to one of four templates. After reviewing the experimental materials, the participants were asked to complete a series of questions, provide demographic information, and indicate their perceptions of the online platform. The experiment lasted approximately 40 min, and the participants were thanked for their participation and debriefed on the true purpose of the study.

**Figure A1:**
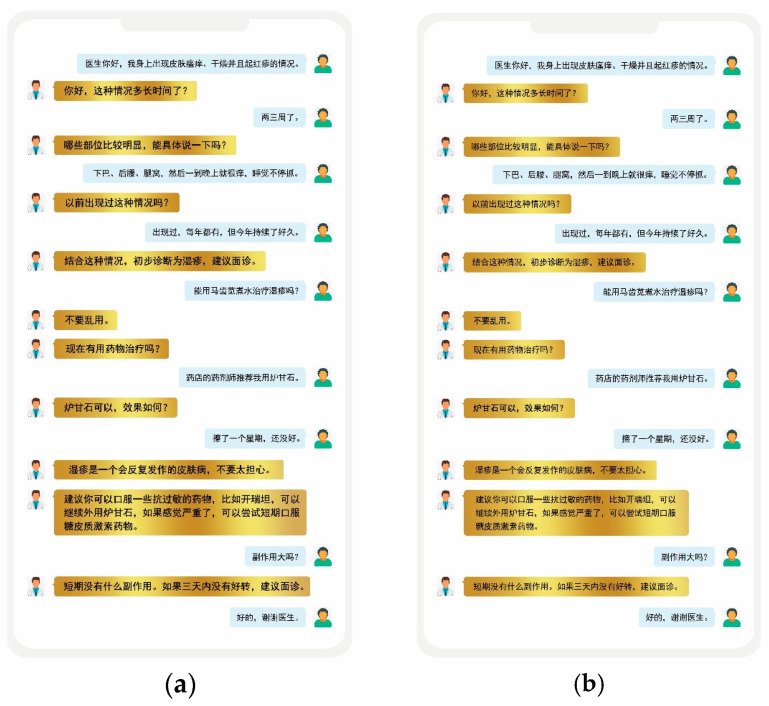

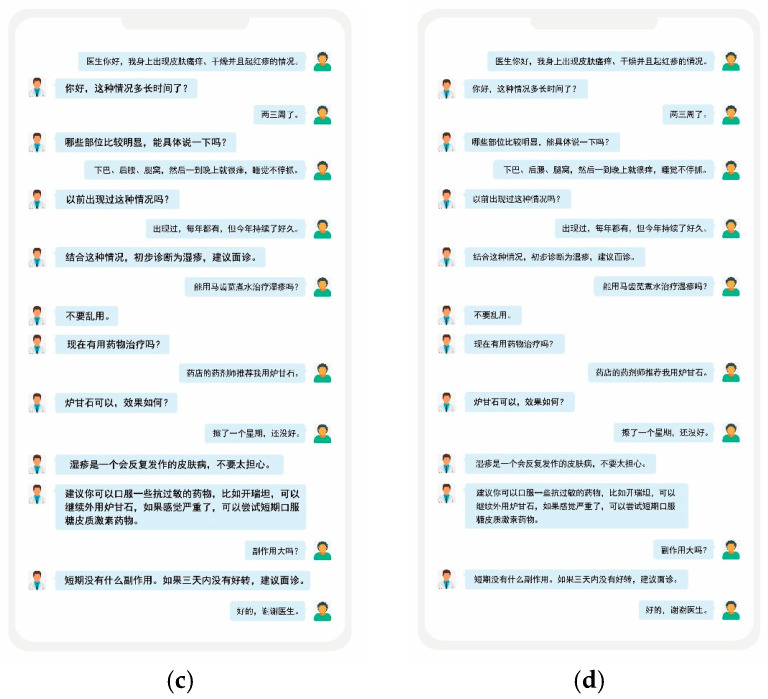
Conversation page design (**a**–**d**).

In UI interface design, bold fonts are easily recognizable, standardized, correct, and dynamic, and they are often used to highlight key information. The font size, the line spacing, and their connotations have a significant impact on reading difficulty and visual fatigue [48]. A font that is too large or too small on a cell phone can make the reader feel uncomfortable. Studies have found that 10, 12, 14, 16, 20, and 22 fonts are preferred for small and medium fonts [49]. Considering the special characteristics of Chinese fonts, we adopted the centering principle and chose the font points of 14 and 16. To test the hypothesis, a web-based physician–patient dialogue template was created, using eczema as a sample dermatological condition. A full between-group experiment was designed to examine the hypothesis, with a 2 (dialogue box color: gold vs. grey) ×2 (font size: large vs. regular) design. The experiment attempted to measure the patients’ perception of physician authority through different designed interfaces and screened which interface design had significance regarding the patients’ perception of physician authority. Therefore, the experiment changed the font size and background color of the physicians’ information in the interface and conducted the experiment by controlling independent variables and moderating variables. The visual stimulus materials were expertly created by a web designer and consisted of four pages, each of which displayed the following attributes:

(1) the physician’s dialogue box was displayed in a gold color with a large font (16-point bold);
(2) the physician’s dialogue box was displayed in a gold color with a regular font (14-point bold);
(3) the physician’s dialogue box was displayed in a grey color with a large font (16-point bold);
(4) the physician’s dialogue box was displayed in a grey color with a regular font (14-point bold);

This is illustrated in Appendix A Figure A1 and Table A1.

### 3.3. Measurement

The data collection was conducted through the use of the survey method. The item perception of authority was closely adapted from Todd DeZoort et al.’s [50] study to develop and provide psychometric assessment of a new authoritarianism scale and was comprised of items sourced from four dimensions of authority, including the general attitudes toward authority, organizational conflict, compliance, and social desirability. The offline willingness to visit item was adapted from Venkatesh et al. [51]. Both the perceived authority and offline willingness to visit items were assessed using a five-point Likert scale, with responses ranging from “completely disagree” (1) to “strongly agree” (5). The nine items utilized in this study are presented in Appendix A Table A2.

### 3.4. Statistical Methods

The experimental data collected in this study were analyzed using SPSS 22.0 software and the PROCESS (version 3.1) plug-in. Descriptive analyses were conducted on both the perceptions of authority and the willingness to visit offline. The relationship between the perceptions of authority and the willingness to visit the clinic was analyzed using Pearson’s coefficient, with the mean of the question items serving as the basis for both. Finally, the mediating role of perceptions of physician authority in the relationship between the dialogue box color and patients’ willingness to visit the clinic and the moderating role of the font size were examined using the PROCESS (version 3.1) plug-in for SPSS. This computational tool, which was developed by Hayes (2013) and Bolin (2014), is a widely accepted standard for analyzing mediating and moderating effects in the social sciences. In accordance with recommended practices, all regression/path coefficients were standardized, and pre-analysis variables were mean-centered.

## 4. Results

### 4.1. Descriptive Statistics and Operational Tests

To examine the potential presence of interaction effects in a sample of four experimental groups, the minimum required sample size was calculated using the G*Power 3.1 software. The calculation indicated that a minimum sample size of 171 participants was necessary for a moderate effect size, as represented by Cohan’s f of 0.25. To ensure adequate power for the analysis, a sample of 240 participants was recruited for the experiment. We employed a randomized design, where these participants were evenly divided into four groups (I to IV), with 60 individuals in each group. The descriptive statistics and correlation coefficients for the key variables are presented in Table 1 and Table 2, respectively. To compare the demographic characteristics between the four experimental groups, an analysis of variance (ANOVA) and Fisher’s exact test were conducted. The results indicated that the distribution of participants across the four groups was not significantly different in terms of age (mean age = 19.47 ± 1.11) or gender (57 males, 183 females), suggesting that the random allocation of participants was effective. Furthermore, no demographic differences were observed between the groups. Finally, a Pearson correlation analysis was performed, which revealed a significant positive correlation between participants’ perceived authority and their opinion of offline visits (r = 0.257, *p* < 0.01).

### 4.2. Mediated Moderation Model Test

The model employed in the analysis was Model 7, which is a mediated model that assesses the influence of the font size (either large or regular) as the independent variable, the dialogue box color (gold or grey) as the moderating variable, the physician’s authority perception as the mediating variable, the patient’s willingness to seek offline medical treatment as the dependent variable, and the covariates of gender and age. To ensure robust results, a bootstrapping technique with 5000 iterations was utilized to detect any indirect effects. In order to investigate the effect of font size on the relationship between the dialogue box color and perceived authority, we applied a regression-based approach to the previously proposed concept of the autonomy penalty.

The results of the regression analysis indicate that a larger font size compared to a regular font size significantly increased the perceptions of physician authority (B = −0.23, SE = 0.04, t = −6.23, *p* < 0.01). Additionally, the use of gold dialogue boxes was found to increase perceptions of physician authority compared to grey dialogue boxes (B = −0.63, SE = 0.04, t = −17.12, *p* < 0.01). Furthermore, perceptions of physician authority were found to have a positive impact on the willingness of participants to visit the clinic (B = 0.40, SE = 0.16, t = 2.45, *p* < 0.05). Demographic factors, such as age and gender, had little effect on the participants’ opinions of physician authority. A bootstrap analysis with 5000 samples revealed that the text size did not have a direct effect on the willingness to visit a physician offline, as the direct effect was not significant (95% CI = (−0.5113, 0.0482)), with perceptions of authority playing a fully mediating role in this relationship (95% CI = (−0.9063, −0.2540)). These results are presented in Table 3.

**Figure 1:**
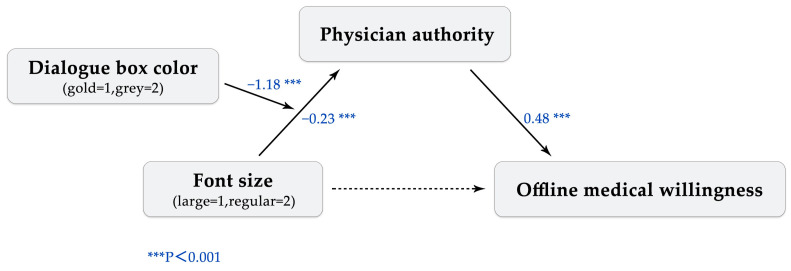
Moderated mediation model.

**Figure 2:**
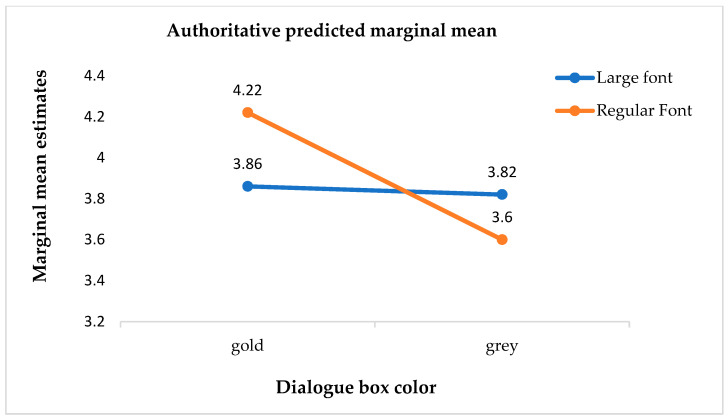
Interaction effect of the font size and dialogue box color on the perception of authority.

Meanwhile, the model results showed that the dialogue box color significantly moderated the effect of the text size on physician authority (B = −1.18, SE = 0.07, t = −16.06, *p* < 0.01), as depicted in Figure 1. Participants who read large-font text (M = 3.86, SD = 0.25) had a significantly lower perception of physician authority compared to those who read regular-font text (M = 4.22, SD = 0.33) in the context of a golden dialogue box. Conversely, in the case of a grey dialogue box, participants who read large-font text (M = 3.82, SD = 0.32) had a significantly higher perception of physician authority compared to those who read regular-font text (M = 3.60, SD = 0.67), as indicated in Figure 2.

## 5. Discussion

This study aimed to delve into the relationship between the design elements of the online physician–patient communication interface and the resulting perceptions and behaviors elicited from patients during online medical consultations. To achieve this, we adopted a between-group experimental design, manipulating two crucial factors, the color of the dialogue box (gold vs. grey) and the font size (large vs. regular), as independent variables. The nuanced findings we derived through this empirical exploration shed light on the multifaceted role design plays in shaping perceptions of expert authority and subsequent behavioral decisions.

The data analysis revealed an intriguing pattern—patients perceived physicians as having higher authority when the dialogue box was gold and the text was in a larger font size compared to the traditional grey dialogue box with a regular font size. This perception of heightened authority manifested as a positive motivator towards seeking offline consultations. Intriguingly, demographic factors such as age and gender had negligible influence over the patients’ perception of a physician’s authority. Furthermore, the dialogue box color was shown to modify the perception of authority created by the text size, adding another layer of complexity to our findings.

However, without supporting our hypothesis, the interface design elements did not have a direct and significant impact on patients’ willingness to move towards offline consultations. Instead, it was the mediating role of the perceived authority that was prominent, emphasizing the paramount importance of perceived authority in driving patient behavior.

In light of these findings, it becomes clear that the visual appeal of the physician–patient interface, including elements such as the color of the dialogue box and font size, can significantly sway the patients’ perception of a physician’s authority online. Our study suggests that using a gold dialogue box coupled with larger text can enhance the authority of the physician in the eyes of the patient. These findings cohere with the existing body of research that suggests brighter colors such as gold are seen as more attractive and visually salient [24].

On the flipside, our results also raise the possibility that the use of color might sometimes attenuate the sense of authority. The somewhat inconsistent effects of font size on patients’ perception of authority [52] might be linked to the initial impression or primacy effect [53,54] caused by the use of color. The combination of a large font size and a gold dialogue box could be construed as overly assertive or perhaps even arrogant [24], leading to a reduction in perceived authority when compared to interfaces employing regular font sizes [55].

Another pivotal finding from our research suggests that the perceived authority of online physicians significantly affects patients’ willingness to seek offline consultations. This aligns with previous research demonstrating that the perception of a physician’s authority online directly influences offline consultation behavior [28,47,56]. It was observed that patients who trusted online physicians and found them psychologically comforting [47,57] were more willing to seek offline consultations [58].

Finally, our study concluded that the design of the communication interface influences patients’ willingness to seek offline consultations through the mediating mechanism of perceived authority, not directly. This aligns with previous studies [27,59] that suggest that the page design mainly affects individuals’ perceptions, which then, in turn, dictate their behavioral responses. The high perceived authority of the physician is reflected in the positive trust in the physician and the positive psychological comfort to the patient himself, and it is this perception that positively influences the patient’s willingness to visit the physician offline. This crucial finding emphasizes the importance of understanding the psychological mechanisms at play in online medical consultations and paves the way for further research in telemedicine interface design.

## 6. Conclusions

The rapid proliferation of digital healthcare underscores the ever-increasing significance of online physician–patient interactions, acting as a cornerstone of contemporary health communication strategies. In response to this evolving paradigm, this research ventured to investigate the subtle yet profound effects of online dialogue box design on patients’ perceptions of a physician’s authoritative presence, which consequently impact their intent to schedule offline consultations. Our findings serve as empirical evidence highlighting the transformative power of meticulously crafted interface design in shaping patients’ perceptions, thereby influencing their behavior to seek offline medical assistance.

This study adds a new dimension to the burgeoning research in online healthcare by focusing on the often-overlooked facet of design. Although the juxtaposition between online and offline healthcare systems has been thoroughly explored, the emphasis on the role of design aesthetics in promoting efficacious online physician–patient communication has largely been neglected.

With respect to the specifics of design impacts, our findings illuminate that visual elements, including the font size and the color of dialogue boxes, can significantly augment the online authority perceived of the physician. However, this authority is not solely a byproduct of design but can also be shaped and enhanced by the communication strategies that physicians employ during online consultations. The intersection between design elements and communication strategies opens an exciting avenue for future research, warranting a more detailed exploration.

Further, our study underscores the intriguing paradox in online healthcare communication. While the perception of authority was found to correlate positively with patients’ willingness to opt for offline consultations, an overemphasis on authority could potentially intimidate patients, thereby serving as a deterrent for seeking offline treatment. Therefore, striking a harmonious balance between asserting authority and fostering an approachable demeanor emerges as a crucial skill for physicians in online settings.

This research offers several pertinent insights, both for platform designers and medical practitioners. Designers of telemedicine interfaces can leverage these findings to understand how the strategic manipulation of design elements can enhance the perceived authority of physicians, thereby creating more effective digital health communication platforms. On the other hand, physicians can harness these insights to modify their online interaction styles, thereby optimizing the potential for transitioning online consultations to offline visits.

In conclusion, this research augments the scholarly discourse on telemedicine interface design, shedding light on the influential role of design aesthetics in modulating patients’ perceptions and subsequent behaviors. However, despite these meaningful contributions, several areas warrant further exploration.

The present study opens up a plethora of opportunities for future research. For instance, researchers could delve deeper into how different combinations of design elements affect patients’ perceptions, exploring potential synergies or conflicts between these elements. Furthermore, research could be extended to explore how these effects vary across different demographic groups, medical conditions, or cultural contexts. Additionally, longitudinal studies could examine how patients’ perceptions and behaviors evolve over time in response to consistent or changing design elements.

Our findings are a testament to the transformative power of design, and we hope that they will inspire a new wave of research dedicated to optimizing the digital physician–patient interface, ultimately paving the way for a more patient-centered and engaging online healthcare ecosystem.

## Figures and Tables

**Table 1:** Summary of descriptive statistics for key variables.

Groups	Gender	Age
Group I (N = 60)	Male = 14	19.42 (±1.03)
Group II (N = 60)	Male = 14	19.23 (±1.11)
Group III (N = 60)	Male = 14	19.55 (±1.15)
Group IV (N = 60)	Male = 15	19.70 (±1.14)
	X2 = 0.07, *p* > 0.05	F (3239) = 1.81, *p* > 0.05

**Table 2:** Correlation coefficients of key variables.

Variable	M	SD	Topic Term	N	Pearson Coefficient
Authority perception	3.72	0.53	6	240	
Offline medical willingness	3.47	1.11	3	240	0.257 **

Notes: ** indicates *p* < 0.01

**Table 3:** Direct and indirect effects of color on BI (N = 240).

Effect	B	Boot SE	Boot LLCI	Boot ULCI
Direct effect	−0.2299	0.1739	−0.5113	0.0482
Indirect effect	−0.4753	0.1945	−0.9063	−0.2540

Note: Boot LLCI, Boot CI lower limit; Boot ULCI, Boot CI upper limit.

## Data Availability

The data that support the findings of this study are available from the corresponding author upon reasonable request.

## Appendix A

**Table A1:** Translation of a conversation between a physician and a patient in the experiment material.

Physician	Patient
	Hello, physician. My skin is itchy, dry, and has a rash.
Hello, how long has this been happening?	
	Two or three weeks
Which parts of your body itch more? Can you point them out?	
	The chin, back, waist, and leg sockets itch at night.
Have you had this complaint before?	
	Yes, every year, but this year has lasted for longer.
In combination with this condition, you were initially diagnosed with eczema, and a face-to-face diagnosis was recommended.	
	Can I use purslane to boil water to treat eczema?
Don’t misuse it. Is there any medication now?	
	The pharmacist at the drugstore recommended calamine.
Calamine is ok. Has there been any improvement?	
	It’s been a week since the medicine was applied, but it hasn’t healed yet.
Eczema is a skin disease that can recur. Don’t worry too much.	
It is recommended that you take some anti-allergic drugs orally, such as Kairuitan, and continue to use calamine externally. If you feel serious discomfort, you can try taking glucocorticoid drugs orally for a short time.	
	Is there any considerable side effect?
There are no side effects in the short term. If you do not get better within three days, you are recommended to consult offline.	
	Ok, thank you, physician.

**Table A2:** Measurement items of constructs.

Construct	Measurement Items
Authoritative Perception	I should do what the physician says, even if I can’t see the reason.
	I should follow the physician’s reasonable requests during treatment.
	I am obligated to follow the physician’s wishes in treatment.
	I have no room to rebel against the physician’s wishes in treatment. *
	It is desirable for me to obey the physician in treatment.
	If the physician asks me to do something, I will do it.
Offline Medical Willingness	I intend to visit this physician in the hospital in the future.
	I predict visiting this physician in the hospital in the future.
	I plan to visit this physician in the hospital in the future.

Note: * is a reverse scoring entry.
